# 

**Published:** 1885-08-20

**Authors:** 


					﻿A RELIABLE	mill |Z	AE?	AA A Z* III C Cl A	A CONCENTRATED
ANTACID.	MILK	1/1"	IVlAVlMtCHA.	fluid ma&nesia.
Particularly indicated in Cholera Infantum. Diarrhoea and Stomach Disorders.
/	THE CHAS H. PHILLIPS CHEMICAL CO., 30 Platt St., N. Y.
VOL. XV. AUGUST 20, 1885. NO. 8.
TSLE
SOUTHERN MEDICAL RECORD:
A MONTHLY JOURNAL OF PRACTICAL MEDICINE.
Terms: $2.00 per Annum, in Alliance; 4 Copies $6.00; Single Copies 20 Cents.
« QUICQUID PRJECIPIES ESTO BREVIS.”
Address J}. C. WORD, M.D., Managing Editor, Atlanta, Ga.
Entered at the Post-Office at Atlanta, as second-class matter.
				

